# Understanding of metal-insulator transition in VO_2_ based on experimental and theoretical investigations of magnetic features

**DOI:** 10.1038/s41598-018-35490-5

**Published:** 2018-11-20

**Authors:** R. Zhang, Q. S. Fu, C. Y. Yin, C. L. Li, X. H. Chen, G. Y. Qian, C. L. Lu, S. L. Yuan, X. J. Zhao, H. Z. Tao

**Affiliations:** 1 School of Physics, Huazhong University of Science and Technology, Wuhan, 430074 P. R. China; 2 State Key Laboratory of Silicate Materials for Architectures, Wuhan University of Technology, Wuhan, 430070 P. R. China

## Abstract

The metal-insulator transition temperature *T*_c_ in VO_2_ is experimentally shown to be almost the same as a magnetic transition temperature *T*_m_ characterized by an abrupt decrease in susceptibility, suggesting the evidence of the same underlying origin for both transitions. The measurement of susceptibility shows that it weakly increases on cooling for temperature range of *T* > *T*_m_, sharply decreases near *T*_m_ and then unusually increases on further cooling. A theoretical approach for such unusual observations in susceptibility near *T*_m_ or below is performed by modeling electrons from each two adjacent V^4+^ ions distributed along V-chains as a two-electron system, which indicates that the spin exchange between electrons could cause a level splitting into a singlet (*S* = 0) level of lower energy and a triplet (*S* = 1) level of higher energy. The observed abrupt decrease in susceptibility near *T*_m_ is explained to be due to that the sample enters the singlet state in which two electrons from adjacent V^4+^ ions are paired into dimers in spin antiparallel. By considering paramagnetic contribution of unpaired electrons created by the thermal activation from singlet to triplet levels, an expression for susceptibility is proposed to quantitatively explain the unusual temperature-dependent susceptibility observed at low temperatures. Based on the approach to magnetic features, the observed metal-insulator transition is explained to be due to a transition from high-temperature Pauli paramagnetic metallic state of V^4+^ions to low-temperature dimerized state of strong electronic localization.

## Introduction

Vanadium dioxide (VO_2_) is generally shown to undergo a metal-to-insulator (M-I) transition at a temperature *T*_c_ ~ 340 K^1,2^, which is accompanied by a simultaneous structural distortion from a high-temperature (*T*) rutile-type tetragonal (R) phase to a low-*T* monoclinic (M) phase^2–5^. This structural distortion as an important focus in M-I transition has also been verified by recent studies^6–8^. Since its discovery more than forty years ago, this transition has been attracting considerable interest for fundamental reasons^9–16^, and for possible applications^7,17–23^. Although various approaches have been proposed^2,9–16,24^, a complete and generally accepted picture of the transition has not yet been developed. It is generally believed that the transition to the insulating state is due to the formation of dimers, each being formed by two electrons from adjacent V^4+^ ions distributed along V-chain direction in spin antiparallel. However, a basic question is still unclear, that is-, whether does the formation of dimers cause a structural distortion to the M phase or does the structural distortion lead to the formation of dimers? This question is studied in the present work by investigations of magnetic features in VO_2_ based on measurements for the temperature dependence of susceptibility and a theoretical approach to electronic state by modeling electrons from each two adjacent V^4+^ ions distributed along V-chain direction as a two-electron system. Magnetic measurements demonstrate that the VO_2_ undergoes a magnetic transition characterized by an abrupt decrease in susceptibility near the M-I transition temperature, and shows Pauli and unusual paramagnetic (PM) behaviors above and below the transition temperature, respectively. Despite structural, electronic, magnetic properties and even multi degrees of freedom coupling involved have been proposed, a generally consensus on unusual behaviors of VO_2_ has not been developed so far^25–27^. The observed different magnetic behaviors are discussed by considering whether the spin exchange exists between electrons from adjacent V^4+^ ions along V-chain direction or not.

## Results

**Figure 1:**
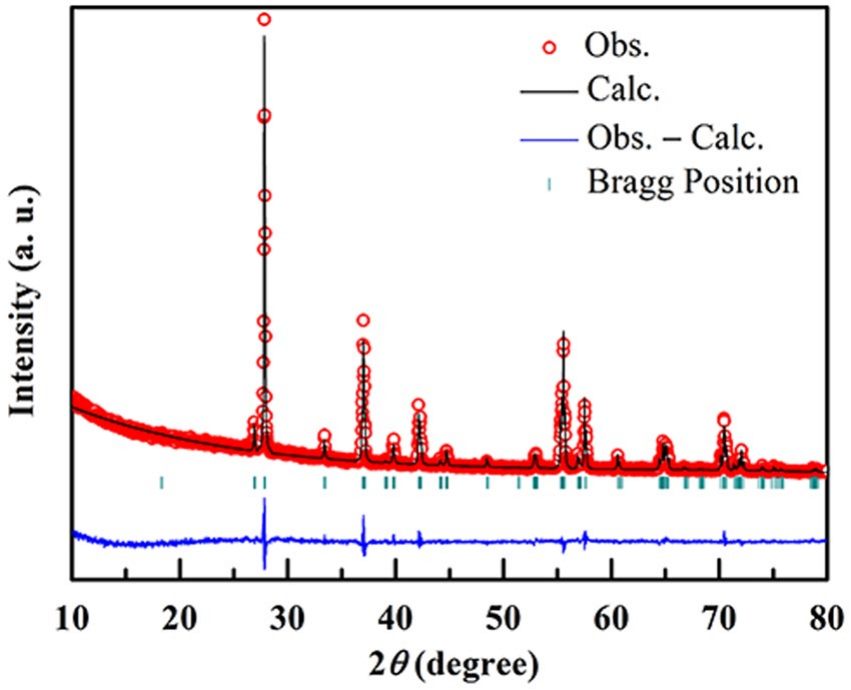
Room-temperature X-ray diffraction pattern and its refinement by using Fullprof 2.0.

A bulk VO_2_ polycrystalline sample is used in the present experimental investigation, which was prepared by hydrothermal method. Stoichiometric quantities of vanadium pentoxide (V_2_O_5_, 99.9%) and oxalic acid (99.5%) with a substance ratio of 1:1.5 were mixed and dissolved in pure water. The mixed liquid was put into high pressure proportion and heated at 180 °C for 48 h. The reacted intermediate VO_2_ (B) powders were annealed at 550 °C for 2 h in vacuum, then pressed into pellets and sintered at 900 °C for 4 h in vacuum to get the final product VO_2_ (M) bulks. Shown in Fig. 1 is a refinement of X-ray diffraction pattern measured at room temperature by using Fullprof 2.0. All the diffraction peaks can be indexed to a M phase of *β* = 122.6° with P2_1_/c space group, and no secondary phase can be detected. The lattice parameters are calculated to be *a* = 0.575048 nm, *b* = 0.452436 nm and *c* = 0.538120 nm, respectively.

**Figure 2:**
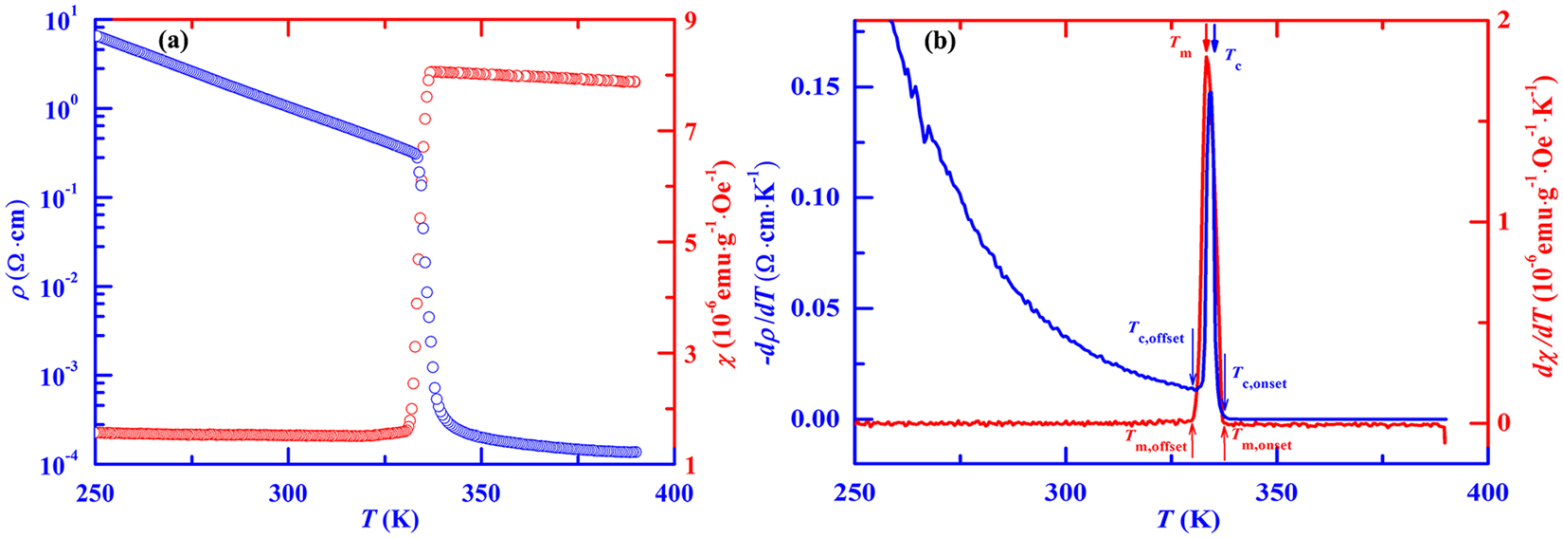
Temperature dependences of (**a**) resistivity (blue circles) and susceptibility (red circles) as well as (**b**) corresponding differential data, $-d\rho /dT$ (blue curve) and $d\chi /dT$ (red curve).

The zero-field resistivity ($\rho$) and susceptibility $(\chi )$ for an applied magnetic field of 1000 Oe as functions of *T* are measured in a commercial physical property measurement system (PPMS, Quantum Design). Because the sample resistance at low temperatures is over range of PPMS, the $\rho -T$ dependence was measured only for the temperature range of *T* > 250 K. In Fig. 2 we display $\rho (T)$ and $\chi (T)$ data measured during cooling process, and corresponding differential curves. A sharp increase in $\rho$ can be found in the $\rho -T$ curve, which is generally explained to be a result of M-I transition. From the differential curve, we determine the M-I transition temperature *T*_c_ to be ~334 K at which $-d\rho /dT$ reaches its maximum, and the onset temperature ${T}_{c,\mathrm{onset}}$ for the occurrence of M-I transition to be ~338 K from which $-d\rho /dT$ starts to increase sharply. On the other hand, an evidence for the occurrence of magnetic transition can be found in the $\chi -T$ curve, which displays an abrupt decrease in *χ*. From the $d\chi /dT$ *vs*. *T* curve, we determine the magnetic transition temperature *T*_m_ to be ~333.5 K at which $d\chi /dT$ reaches its maximum, and the onset temperature *T*_m,onset_ for the occurrence of magnetic transition to be ~338 K from which $d\chi /dT$ sharply increases. It is essential to note that both the *T*_c_ and *T*_m_, or *T*_c,onset_ and *T*_m,onset_, are experimentally confirmed to be almost the same, suggesting the evidence of same origin for both the M-I and magnetic transitions. Therefore, understanding the magnetic feature involving in VO_2_ is helpful to find a clue to reveal the nature of M-I transition. It should be mentioned that the susceptibility as a function of temperature was also measured at various constant applied magnetic fields up to 9 T, no effect of magnetic field to magnetic transition was found. Because of this fact, in Fig. 2 we only display the $d\chi /dT$ *vs*. *T* curve measured at the 1000 Oe field.

**Figure 3:**
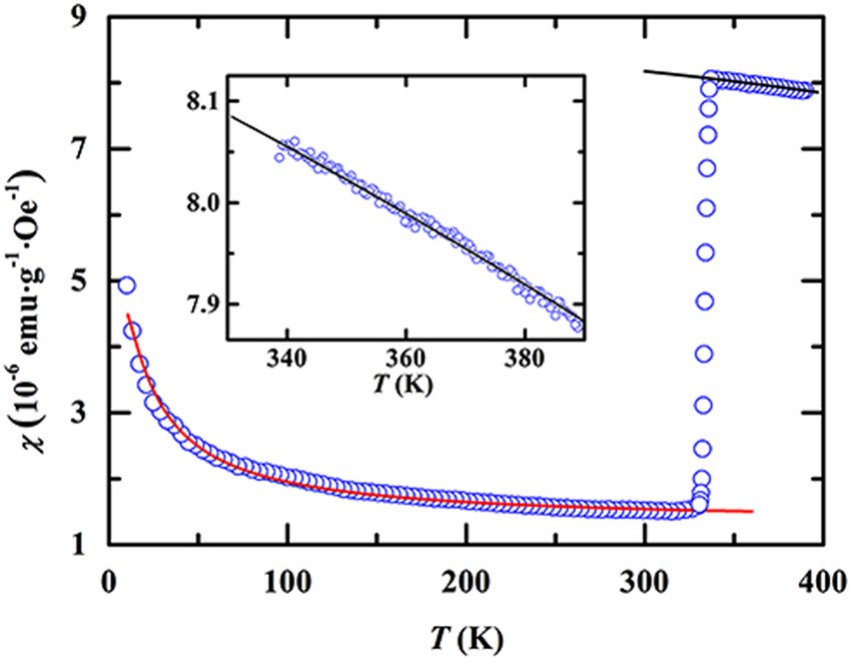
Temperature dependence of susceptibility measured in VO_2_. Both solid black and red lines for high-*T* and low-*T* ranges are curves calculated from Eqs (4) and (6), respectively. Inset is a comparison between high-*T* experimental data and curve calculated by Eq. (4).

The VO_2_ is of paramagnetism for the whole range of temperature covering from high-*T* metallic to low-*T* insulating states, which can be confirmed through the measurement of hysteresis loop in which no magnetic hysteresis becomes visible at various fixed temperatures from 370 K to 10 K^28^. Nevertheless, the experimental fact of magnetic transition displayed in Fig. 2 proposes that the PM behavior at *T* > *T*_m_ should be different from that at *T* < *T*_m_. In Fig. 3 we display the *χ vs*. *T* curve measured in the field of 1000 Oe during cooling process for a wide range of temperature from 390 K to 10 K. It can be seen that the susceptibility increases weakly on cooling for the range of *T* > *T*_m,onset_, passing through the transition region, it no longer decreases but unusually increases on cooling from a temperature denoted by *T*_m,offset_ ~ 330 K.

## Discussion

**Figure 4:**
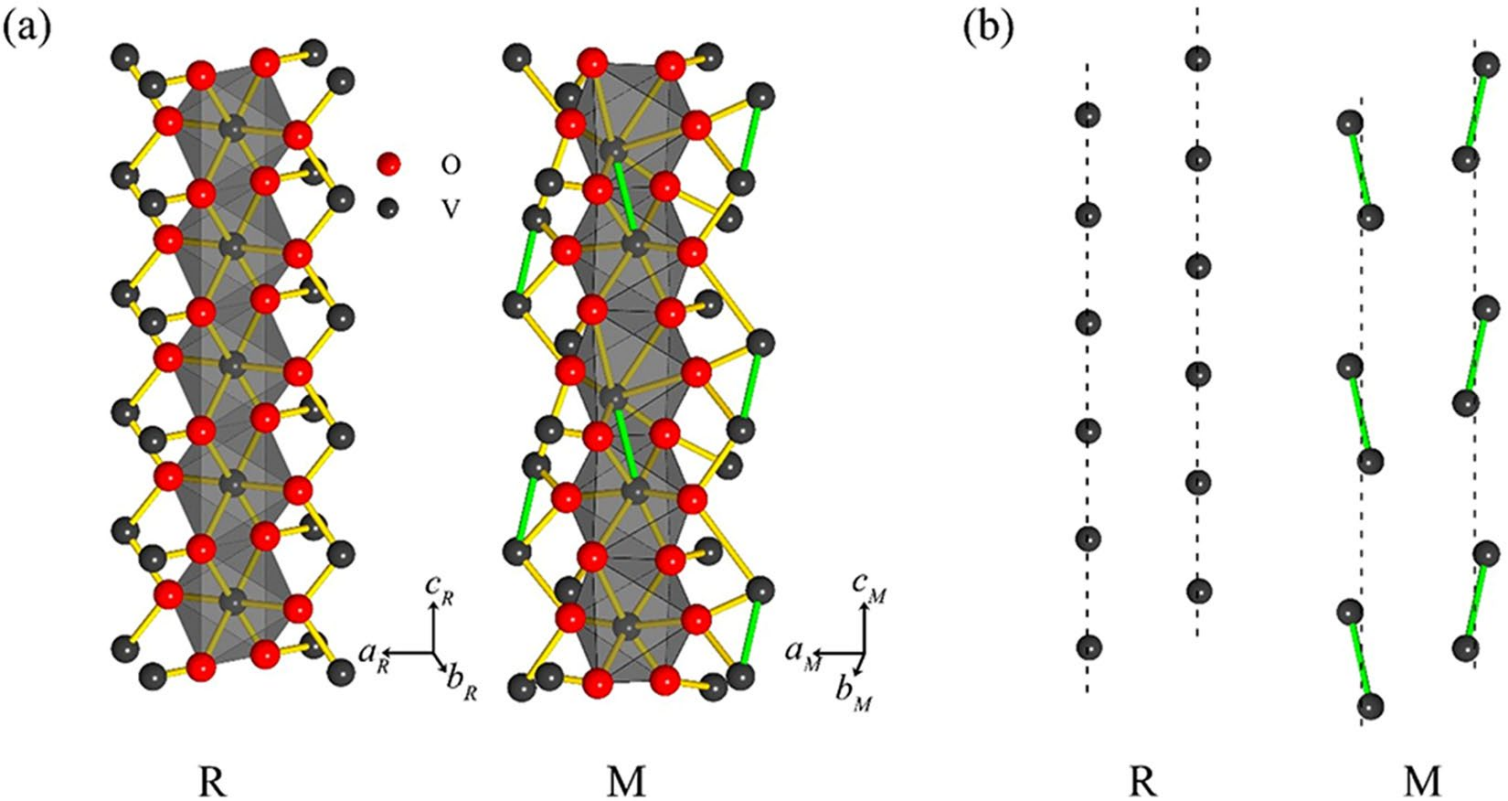
Illustrations of (**a**) crystalline structures of high-*T* R and low-*T* M phases in VO_2_, and (**b**) two parallel straight chains each consisting of V^4+^ ions in the R phase and two parallel zigzag chains each consisting of V-V dimers in the M phase.

In order to explain observed different PM behaviors, here we perform a theoretical approach to electronic states in VO_2_. As well known, a striking feature in crystalline structure of VO_2_ illustrated in Fig. 4(a) is the existence of parallel chains consisting of V^4+^ ions. In the high-*T* R phase, the periodical distribution of V^4+^ ions forms parallel straight chains along the ${c}_{R}$ axis, while in the low-*T* M phase, the V^4+^ ions distribute periodically in zigzags along the ${c}_{M}$ axis^29^, as illustrated in Fig. 4(b). As a general case, we can view each chain as a chain consisting of ion pairs periodically distributed along the chain direction, where each ion pair contains two V^4+^ ions. Note that each V^4+^ ion only has a *d*-electron, therefore, one ion pair has two electrons so that it can be viewed as a two-electron system. According to Heisenberg model^30^, the Hamiltonian of the two-electron system can be written in the form

1 $$\hat{H}({\mathop{r}\limits^{\rightharpoonup }}_{1},\,{\mathop{r}\limits^{\rightharpoonup }}_{2})={\hat{h}}_{1}+{\hat{h}}_{2}+\hat{H}^{\prime} (1,\,{\rm{2}}),$$

where$\,{\hat{h}}_{i}=\,-\frac{{\hslash }^{2}}{2m}{\nabla }_{i}^{2}+V({\mathop{r}\limits^{\rightharpoonup }}_{i})$ is one-electron Hamiltonian of electron *i*,$\,V({\mathop{r}\limits^{\rightharpoonup }}_{i})\,$is a potential experiencing by electron *i*, and $\hat{H}^{\prime} (1,\,{\rm{2}})=-\,2J{\hat{S}}_{1}\cdot {\hat{S}}_{2}$. is the spin exchange between electrons 1 and 2 with exchange constant *J*.

**Figure 5:**
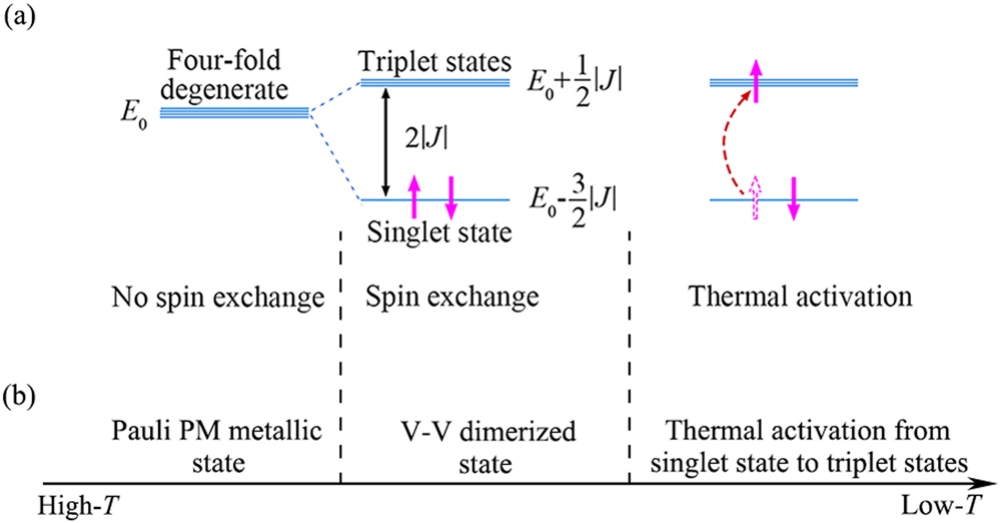
Illustrations of (**a**) the level splitting due to the spin exchange and the electron excitation due to thermal activation and (**b**) the change of electronic states with temperature.

As shown in appendix, if no spin exchange exists between electrons, the two-electron system is of four-fold degenerate with a same energy ${E}_{0}$, while once the spin exchange appears between electrons, the energy level of four-fold degenerate would become splitting into a singlet (*S* = 0) level and a triplet (*S* = 1) levels. The theoretical approach presents the energy

2 $${E}^{S}={E}_{1}={E}_{0}+\frac{3}{2}J$$

for the singlet state, and

3 $${E}^{T}={E}_{2}={E}_{3}={E}_{4}={E}_{0}-\frac{1}{2}J$$

for triplet states. Since the spin exchange term in Eq. (1) is the scalar product of two vector spin operators, it should favor parallel spins if *J* is positive and antiparallel if *J* is negative. For the present system, the antiparallel (*S* = 0) spin alignment of two electrons is more favorable than the parallel (*S* = 1), meaning that *J* should be negative. The present approach therefore indicates that the spin exchange between electrons can cause a splitting of the four-fold degenerate level into a singlet (*S* = 0) state of lower energy and triplet (*S* = 1) states of higher energy with energy separation ${\rm{\Delta }}E={E}^{T}-{E}^{S}=2|J|$, as sketchy illustrated in Fig. 5(a). In Fig. 5(b) we also display illustration of change of electronic states with temperature as discussed below.

Based on the above theoretical approach, we can discuss on magnetic features and present interpretations of experimental observations in VO_2_. In the high-*T* R phase, electrons from two adjacent V^4+^ ions are separated in a larger distance so that the spin exchange between electrons can be neglected. In this case, electrons should show behavior similar to near-free electrons. This should be the reason why the VO_2_ shows metallic behavior at *T* > *T*_c,onset_. Because of the near-free behavior of electrons, the magnetic behavior should be described to the Pauli PM theory similar to that seen commonly in simple metals. According to Pauli PM theory^31^, the temperature dependence of susceptibility can be expressed in the form

4 $${\chi }_{P}={\chi }_{P}^{0}\{1-\frac{{\pi }^{2}}{12}{(\frac{{k}_{B}T}{{E}_{F}^{0}})}^{2}\},$$

where ${\chi }_{P}^{0}=Na$, $a=\frac{3}{2}\frac{{\mu }_{B}^{2}}{{E}_{F}^{0}}$, *N* is the number of “free” electrons,${E}_{F}^{0}$ the Fermi energy at $T\to 0{\rm{K}}$, and ${\mu }_{B}$ the Bohr magneton. In Fig. 3 and its inset, we present a comparison between data measured at high temperatures and curve calculated in terms of Eq. (4) by using parameters of ${\chi }_{P}^{0}=8.6\times {10}^{-6}$ (emu·g^−1^·Oe^−1^) and ${E}_{F}^{0}=105.6$ (meV). It can be seen that the Pauli PM theory yields an excellent agreement with susceptibility data measured at *T* > *T*_m,onset_. The experimental facts of Pauli paramagnetism at *T* > *T*_m,onset_ and same values in both *T*_c,onset_ and *T*_m,onset_ provide strong support to high-*T* phase above *T*_m,onset_ as a near-free electron system.

On cooling to *T*_m,onset_ or below, the spin exchange appears between electrons from two adjacent V^4+^ ions. According to the above approach, this exchange causes the level splitting into the singlet and triplet levels. From the viewpoint of energy, the singlet level is more favorable for the occupation of electrons. Therefore, if two electrons from adjacent V^4+^ ions along the chain direction stay on the singlet level, the two electrons must be paired in spin antiparallel. It means that the singlet state is a state in which the dimers are formed by pairing V^4+^ ions in spin antiparallel. We, therefore, propose the spin exchange between electrons to be the reason for the formation of dimers in spin antiparallel. On the other hand, associated with the formation of dimers, the straight chain consisting of “free” V^4+^ ions would be distorted into a zigzag chain. It implies that the spin exchange between electrons is the reason causing a structural distortion from high-*T* R phase characterized by straight chains consisting of V^4+^ ions to low-*T* M phase characterized by zigzag chains consisting of dimers.

Owing to that each dimer contains two electrons, associated with the formation of dimers, the number of “free” electrons would change from *N* at *T* > $(N-2{N}_{d})$ at *T* < *T*_m,onset_, where *N*_d_ is the number of dimers. Clearly, *N*_d_ should be zero at *T* > *T*_m,onset_, but increases substantially on cooling from *T*_m,onset_. On the other hand, each dimer has zero spin and cannot contribute to susceptibility. Therefore, the Pauli PM susceptibility would change approximately from *Na* at *T* > *T*_m,onset_ to $(N-2{N}_{d})a$ at *T* < *T*_m,onset_. Because of the substantial increase in *N*_d_ on cooling from *T*_m,onset_, the susceptibility would show an abrupt decrease as seen in experiments. It means that the observed magnetic transition is due to a transition from Pauli PM state of “free” V^4+^ ions at *T* > *T*_m,onset_ to a singlet state at *T* < *T*_m,onset_ in which V^4+^ ions along the chain direction are paired into dimers in spin antiparallel, a result predicted by the above approach. One can also notice that the abrupt decrease in *χ* only maintains to a temperature denoted by *T*_m,offset_ ~ 330 K, meaning that the formation of dimers occurs mainly in a narrow range of *T*_m,offset_ < *T* < *T*_m,onset_ and the number of dimers tends to be a *T*-independent constant *N*_d0_ on further cooling from *T*_m,offset_.

The above approach can be further confirmed by analyzing the *χ vs*. *T* dependence observed below *T*_m,offset_. As indicated in Fig. 3, the susceptibility is no longer decreased but unusually increased on cooling from *T*_m,offse*t*_. The observed unusual increase in *χ* cannot be explained by Pauli PM theory which predicts an almost *T*-independent susceptibility at low temperatures. Although Curie’s law predicts a variation of *χ* inversely with *T*, the question is what kind of PM entities with magnetic moments responsible for the observed low-*T* paramagnetism. Below *T*_m,offset_ the dimers are formed by spin pairing of adjacent V^4+^ ions in antiparallel, so that each dimer has no magnetic moment. Therefore, the dimers cannot contribute to the observed low-*T* paramagnetism, unless that each dimer is phenomenologically assumed to be formed by spin pairing of two V^4+^ ions at a *T*-dependent angle^28^.

Here, we demonstrate that the observed variation of *χ* inversely with *T* can be explained by considering the contribution to Curie PM susceptibility from unpaired electrons created due to the thermal activation from singlet to triplet levels. According to the above approach, the spin exchange causes the level splitting into the singlet and triplet levels. At finite temperatures, it is likely that electrons in the singlet level could be thermally excited to the level of the triplet states, resulting in the appearance of unpaired electrons with number $\propto {N}_{d0}{e}^{-2|J|/{k}_{B}T}$, as sketchy illustrated in Fig. 5. These unpaired electrons with magnetic moments can contribute to Curie-like PM susceptibility. In the Curie’s law, the Curie parameter *C* is proportional to the number of PM ions, therefore, one has $C\propto {N}_{d0}{e}^{-2|J|/{k}_{B}T}$ for the present case. We then write the susceptibility caused by unpaired electrons created due to thermal activation in the form:

5 $${\chi }_{C}=\frac{B{e}^{-2|J|/{k}_{B}T}}{T},$$

where $B\propto {N}_{d0}$ is a *T*-independent constant. Besides those thermally activated electrons, it is likely for the presence of “free” electrons from residual V^4+^ ions which don’t participate in the formation of dimers. As seen at *T* > *T*_m,onset_, these “free” electrons should show Pauli PM behavior with susceptibility approximately expressed by ${\chi }_{P}^{0}=(N-2{N}_{d0})a$. The total susceptibility is then expressed by a sum of co-contributions from two kinds of electrons, that is-

6 $$\chi ={\chi }_{P}^{0}+\frac{B{e}^{-2|J|/{k}_{B}T}}{T}.$$

To support this approach, here we calculate the *χ vs*. *T* curve in terms of Eq. (6) by using $${\chi }_{P}^{0}={\rm{1}}\,{\rm{.}}\,{\rm{32}}\times {\rm{1}}{0}^{-{\rm{6}}}$$$$({\rm{emu}}\cdot {{\rm{g}}}^{-{\rm{1}}}\cdot {{\rm{Oe}}}^{-{\rm{1}}})$$, $$B=6.8\times {\rm{1}}{0}^{-5}({\rm{emu}}\cdot K\cdot {{\rm{g}}}^{-{\rm{1}}}\cdot {{\rm{Oe}}}^{-{\rm{1}}})$$ and $$2|J|/{k}_{B}=7.5$$(K). The calculated curve is displayed by red solid line in Fig. 3. It can be found that Eq. (6) yields excellent agreement with the experimental data for the whole low-*T* range below *T*_m,offset_. Note that $${\chi }_{P}^{0}={\rm{1}}.{\rm{32}}\times {\rm{1}}{0}^{-{\rm{6}}}({\rm{emu}}\cdot {{\rm{g}}}^{-{\rm{1}}}\cdot {{\rm{Oe}}}^{-{\rm{1}}})\propto N-2{N}_{d0}$$ below *T*_m,offset_ and $${\chi }_{P}^{0}=8.6\times {\rm{1}}{0}^{-{\rm{6}}}({\rm{emu}}\cdot {{\rm{g}}}^{-{\rm{1}}}\cdot {{\rm{Oe}}}^{-{\rm{1}}})\propto N$$ above *T*_m,onset_, respectively, their ratio gives an estimation of $$2{N}_{d0}/N$$ to be ~85%. It means that in the low-*T* M phase ~85% V^4+^ ions are paired into dimers, while the remainder ~15% V^4+^ ions as “free” ions are still resided in the system.

Once the above magnetic features are accepted, it is reasonable to explain the electronic transport behavior observed in VO_2_. In the high-*T* R phase, because of no spin exchange existing between electrons, electrons would be of near-free behavior and hence the system shows metallic behavior. On cooling to *T*_m,onset_ or below, the spin exchange between electrons from two adjacent V^4+^ ions leads to the formation of V-V dimers, where each two adjacent V^4+^ ions become one V-V dimer by pairing in spin antiparallel. Because of the antiparallel pairing, electrons would be strongly localized, leading to low-*T* insulating behavior. The observed M-I transition is therefore explained to be due to a transition from high-*T* Pauli PM state of V^4+^ ions to low-*T* dimerized state. On the other hand, because both the M-I and magnetic transitions are all related to the formation of V-V dimers, this is the reason why the system undergoes simultaneous M-I and magnetic transitions at almost the same temperature.

## Conclusions

**Figure 6:**
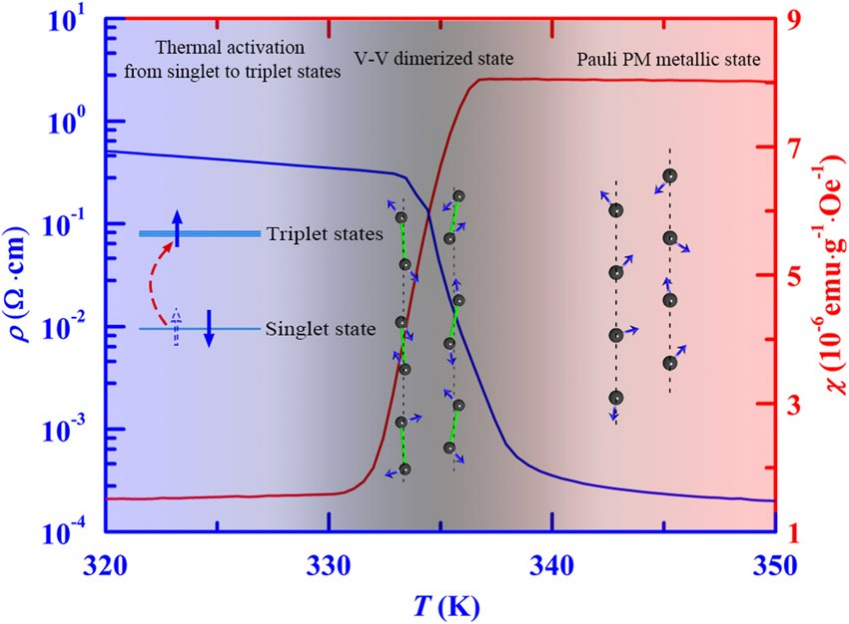
Illustrations of main conclusions obtained in the present work.

In summary, we have shown that the metal-insulator transition in VO_2_ in origin is closely related to the magnetic transition characterized by an abrupt decrease in susceptibility. Based on experimental investigations of susceptibility and the theoretical approach by modeling electrons from two adjacent V^4+^ ions distributed along V-chains as a two-electron system, it is concluded that there is a feature temperature *T*_m_ to distinguish between high-*T* and low-*T* phases. At temperatures slightly higher than *T*_m_, no spin exchange exists between electrons from adjacent V^4+^ ions, as a result, the system shows metallic and Pauli PM behavior. Near *T*_m_, because of the spin exchange, the system enters into the singlet state in which electrons from adjacent V^4+^ ions along the chain direction are paired into dimers in spin antiparallel, resulting in a transition to insulating state and an abrupt decrease in *χ*. At temperatures slightly lower than *T*_m_, the contribution to PM behavior from some unpaired electrons created by the thermal activation from singlet to triplet levels lead to unusual temperature-dependent susceptibility at low temperatures. These main conclusions are summarized in Fig. 6.

## Electronic supplementary material

Supplementary Information
