# Supplementary material for: Understanding of metal-insulator transition in VO2 based on experimental and theoretical investigations of magnetic features
Source: Sci Rep. 2018 Nov 20;8:17093. doi: 10.1038/s41598-018-35490-5 (PMC6244010; doi:10.1038/s41598-018-35490-5)
Supplement: Supplementary file 1 — Supplementary Information [file 41598_2018_35490_MOESM1_ESM.docx]

**Supporting Information**

**Understanding of metal-insulator transition in VO_2_ based on experimental and theoretical investigations of magnetic features**

R. Zhang^1,a)^, Q. S. Fu^1,a)^, C. Y. Yin ^1^, C. L. Li^1^, X. H. Chen^1^, G. Y. Qian^1^, C. L. Lu^1^, S. L. Yuan^1,b)^, X. J. Zhao^2^ & H. Z. Tao^2^

^1^*School of Physics, Huazhong University of Science and Technology, Wuhan 430074, P. R. China*

^2^*State Key Laboratory of Silicate Materials for Architectures, Wuhan University of Technology, Wuhan 430070, P. R. China*

^a)^ *R. Zhang and Q. S. Fu contributed equally to this work.*

^b)^*Author to whom correspondence should be addressed:* [yuansl@hust.edu.cn](mailto:yuansl@hust.edu.cn)

**Contents**

For the two-electron system, if no spin exchange exists between electrons, its Hamiltonian is a sum of the one-electron Hamiltonians, and the corresponding Schrodinger equation becomes

$\hat{H}_{0}\psi(1,2)=(\hat{h}_{1}+\hat{h}_{2})\psi(1,2)=E_{0}\psi(1,2)$. (S1)

The eigenfunctions of the two-electron system can be constructed by a linear combination of and,

,

,

where and are eigenfunctions of electron *i* belonging to the eigenvalues *E_a_* and *E_b_*, respectively, satisfying and . It is noticed the $\psi^{A}(1,2)$ is antisymmetric while the $\psi^{S}(1,2)$ is symmetric because of $\psi^{A}(1,2)=-\psi^{A}(2,1)$and $\psi^{S}(1,2)=\psi^{S}(2,1)$ under permutations of two electrons. The Pauli exclusion principle requires that the total wave function is antisymmetric. This can be ensured by a product of coordinate and spin functions owing to that a permutation of electrons corresponds to a permutation of both the spatial and the spin variables.

Each electron has two spin states denoted by $\alpha$ and $\beta$ corresponding to cases of spin up and down, respectively. Appropriate linear combinations of the four spin states of two electrons can be used to construct the spin functions of the two-electron system as follows:

$\chi_{s1}(1,2)=\frac{1}{\sqrt{2}}\left[\alpha(1)\beta(2)-\alpha(2)\beta(1)\right]$,

$\chi_{s2}(1,2)=\alpha(1)\alpha(2)$,

$\chi_{s3}(1,2)=\frac{1}{\sqrt{2}}\left[\alpha(1)\beta(2)+\alpha(2)\beta(1)\right]$,

$\chi_{s4}(1,2)=\beta(1)\beta(2)$.

For each individual electron one has$\hat{S}_{i}^{2}=\frac{1}{2}(\frac{1}{2}+1)=\frac{3}{4}$, the total spin therefore satisfies$\hat{S}^{2}=(\hat{S}_{1}+\hat{S}_{2})^{2}=\frac{3}{2}+2\hat{S}_{1}\cdot\hat{S}_{2}$. Using$\hat{S}_{x}\alpha=\frac{1}{2}\beta\begin{matrix} , & \hat{S}_{x}\beta=\frac{1}{2}\alpha\end{matrix}$,$\hat{S}_{y}\alpha=\frac{i}{2}\beta\begin{matrix} , & \hat{S}_{y}\beta=-\frac{i}{2}\alpha\end{matrix}$,$\hat{S}_{z}\alpha=\frac{1}{2}\alpha$and $\hat{S}_{z}\beta=-\frac{1}{2}\beta$, it is easily shown that $\hat{S}_{1}\cdot\hat{S}_{2}\chi_{s1}(1,2)=-\frac{3}{4}\chi_{s1}(1,2)$ and $\hat{S}_{1}\cdot\hat{S}_{2}\chi_{sk}(1,2)=\frac{1}{4}\chi_{sk}(1,2)$ $(k=2,3,4)$. One then has $\hat{S}^{2}\chi_{s1}(1,2)=(\frac{3}{2}+2\hat{S}_{1}\cdot\hat{S}_{2})\chi_{s1}(1,2)=0$ for the spin state$\chi_{s1}(1,2)$ and $\hat{S}^{2}\chi_{sk}(1,2)=(\frac{3}{2}+2\hat{S}_{1}\cdot\hat{S}_{2})\chi_{sk}(1,2)=2\chi_{sk}(1,2)$ for the other three spin states. Bearing in mind $\hat{S}^{2}\chi_{sk}(1,2)=S(S+1)\chi_{sk}(1,2)$, the spin function$\chi_{s1}(1,2)$ therefore describes a spin singlet state of *S* = 0, while the other three spin functions$\chi_{sk}(1,2)$ $(k=2,3,4)$ describe spin triplet states of *S* = 1 with three z-components using $\hat{S}_{z}\chi_{sk}(1,2)=(\hat{S}_{1z}+\hat{S}_{2z})\chi_{sk}(1,2)=M_{s}\chi_{sk}(1,2)$, where *M_S_* = 1, 0 and -1 for *k* = 2, 3 and 4, respectively. Note that the$\chi_{s1}(1,2)$ changes sign when the spins of the two electrons are interchanged, while the $\chi_{sk}(1,2)$ $(k=2,3,4)$ does not. To satisfy the antisymmetric requirement, we therefore construct the following four functions as the total wavefunctions of the two-electron system, that is-,

$\psi_{1}(1,2)=\psi^{S}(1,2)\chi_{s1}(1,2)$,

$\psi_{2}(1,2)=\psi^{A}(1,2)\chi_{s2}(1,2)$,

$\psi_{3}(1,2)=\psi^{A}(1,2)\chi_{s3}(1,2)$,

$\psi_{4}(1,2)=\psi^{A}(1,2)\chi_{s4}(1,2)$.

The energy of the two-electron system can be calculated from

$E_{0}=\left\langle\psi_{i}(1,2)\left|\hat{h}_{1}+\hat{h}_{2}\right|\psi_{i}(1,2)\right\rangle$. (S2)

Since the operator $\hat{h}_{1}+\hat{h}_{2}$ does not depend on spin, the energy can be expressed as$E_{0}^{S}=\left\langle\psi^{s}(1,2)\left|\hat{h}_{1}+\hat{h}_{2}\right|\psi^{s}(1,2)\right\rangle$and$E_{0}^{T}=\left\langle\psi^{A}(1,2)\left|\hat{h}_{1}+\hat{h}_{2}\right|\psi^{A}(1,2)\right\rangle$for singlet and triplet states, respectively. Using and , it is easily shown that the two-electron system is of four-fold degenerate due to same energy, i.e., $E_{0}=E_{a}+E_{b}$, for the four states described by$\psi_{k}(1,2)$ $(k=1,2,3,4)$.

The situation is different, if the spin exchange exists between electrons. In this case, the Hamiltonian of the two-electron system should be that indicated by Eq. (1), and the energy *E_k_* corresponding to the state described by $\psi_{k}(1,2)$ $(k=1,2,3,4)$ can be calculated from

$E_{k}=\left\langle\psi_{k}(1,2)\left|\hat{h}_{1}+\hat{h}_{2}\right|\psi_{k}(1,2)\right\rangle+\left\langle\psi_{k}(1,2)\left|-2J\hat{S}_{1}\cdot\hat{S}_{2}\right|\psi_{k}(1,2)\right\rangle$. (S3)

Since $\left\langle\psi_{k}(1,2)\left|\hat{h}_{1}+\hat{h}_{2}\right|\psi_{k}(1,2)\right\rangle=E_{0}$ and the spin exchange term only contains spin variables, the energy *E_k_* can be then simply expressed as

$E_{k}=E_{0}+\left\langle\chi_{sk}(1,2)\left|-2J\hat{S}_{1}\cdot\hat{S}_{2}\right|\chi_{sk}(1,2)\right\rangle$. (S4)

Using $\hat{S}_{1}\cdot\hat{S}_{2}\chi_{s1}(1,2)=-\frac{3}{4}\chi_{s1}(1,2)$and $\hat{S}_{1}\cdot\hat{S}_{2}\chi_{sk}(1,2)=\frac{1}{4}\chi_{sk}(1,2)$ $(k=2,3,4)$, we get the energy

$E^{S}=E_{1}=E_{0}+\frac{3}{2}J$ (S5)

for the singlet state, and

$E^{T}=E_{2}=E_{3}=E_{4}=E_{0}-\frac{1}{2}J$ (S6)

for triplet states.
